# Five-in-one surgery: an integrated approach for chronic Monteggia fracture in children

**DOI:** 10.3389/fsurg.2026.1797510

**Published:** 2026-03-13

**Authors:** Nan Yang, Hui Qin

**Affiliations:** Department of Orthopedics, Shanghai Sixth People's Hospital, Shanghai Jiao Tong University School of Medicine, Shanghai, China

**Keywords:** chronic Monteggia fracture, external fixator, five-in-one surgery, *Henry* approach, redislocation

## Abstract

**Introduction:**

To evaluate the clinical efficacy of the five-in-one surgical approach-comprising *Henry* approach debridement of the humeroradial joint, proximal ulnar osteotomy with lengthening and posterior angulation, hinged external fixator combined with ≥2 K-wire fixation of the osteotomy site, suture repair of the anterior joint capsule, and anterior plaster slab immobilization—in the treatment of chronic Monteggia fractures in children.

**Methods:**

From January 2023 to January 2025, 17 children with Bado Type I chronic Monteggia fractures underwent the five-in-one surgery, with a mean follow-up of 15.3 months (range, 12–36 months).

**Results:**

Anatomical reduction of the radial head was achieved and maintained in 15 patients, and all ulnar osteotomies achieved bony union. Two patients experienced redislocation of the radial head, resulting in a redislocation rate of 11.8%. Significant improvement was observed in elbow flexion (122.4 ± 12.5° vs. 126.5±6.1°, *p* = 0.030) and extension (−5.3 ± 7.2° vs. −2.9 ± 5.3°, *p* = 0.027) postoperatively, with no changes in pronation/supination. No serious complications occurred during the study.

**Discussion:**

The five-in-one protocol effectively restores radiocapitellar stability and elbow function in children with Bado Type I chronic Monteggia fractures.

## 1 Introduction

Monteggia fracture is a rare forearm injury in children, accounting for approximately 1% of all pediatric forearm fractures (1), and is typically characterized by a fracture of the ulna combined with radial head dislocation. Since ulnar fractures in children may present only as plastic deformation or angulation without obvious displacement, approximately 50% of Monteggia fractures are initially missed (2, 3), which progresses to chronic injuries diagnosed months or even years after trauma. Therefore, radial head dislocation should be routinely screened in children with forearm injuries—especially those involving the ulna—to avoid missed diagnosis.

Clinically, limited elbow mobility and valgus deformity are the main presentations, and diagnosis is usually confirmed by X-ray, elbow CT and MRI examinations are also helpful in assessing the extent of radial head dislocation and soft tissue lesions. The Bado classification is the most widely used typing system for Monteggia fractures (4), with Type I (anterior radial head dislocation with ulnar shaft fracture) being the most frequent, accounting for 75%–85% of pediatric cases (5). Injuries diagnosed more than 4 weeks after occurrence are termed chronic Monteggia fractures (6), which may be complicated by nerve injury and heterotopic ossification in addition to typical manifestations of radiocapitellar joint dislocation, limited elbow ROM and pain (7–9). Closed reduction often fails and surgical intervention is required (10). Open reduction of the humeroradial joint combined with ulnar osteotomy is the most frequently performed surgical method (11, 12), while other approaches such as combined ulnar and radial osteotomy have been reported but are less clinically applied (13, 14).

**Figure 1:**
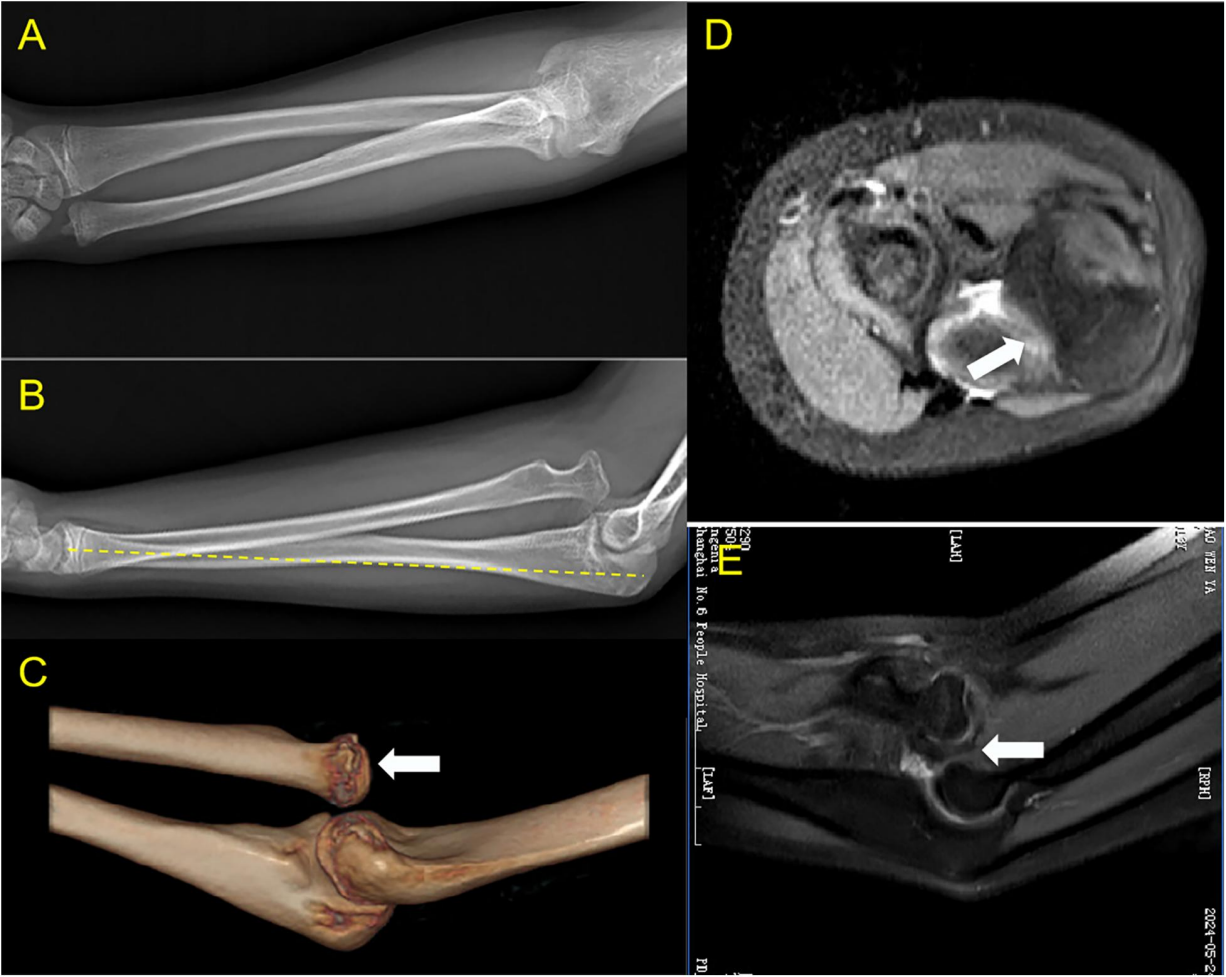
Characteristics of chronic Monteggia fracture: **(A,B)** mismatch in length between the ulna and radius, with the radius relatively longer; anterior angulation of the ulna, presenting as a “bow sign”; **(C,D,E)** radial head dislocated outside the joint capsule, with interposed capsular scar tissue between the radial head and capitellum; deformation of the radial notch of the ulna; alteration of the articular surfaces of the radial head and capitellum.

The surgical outcome of chronic Monteggia fractures is closely associated with the duration of dislocation: better outcomes are achieved in patients with a shorter interval from injury to surgery, while prolonged dislocation is associated with poor prognostic certainty and increased risks of postoperative complications such as radial head redislocation and ulnar nonunion (15, 16). The pathological changes of chronic Monteggia fractures become more severe with prolonged dislocation, including joint capsule contracture, intra-articular scarring, ulnar angulation deformity, relative radial over-lengthening, and articular surface degeneration, which pose great challenges to surgical treatment (5, 14) (Figure 1). To address the uncertainty in treating chronic Monteggia fractures, we propose the five-in-one surgery with the aim of improving therapeutic outcomes.

## 2 Materials

From January 2023 to January 2025, 17 children with chronic Monteggia fractures (all Bado Type I) were treated at our institution. There were 13 males and 4 females, with an age range of 4–15 years (mean 9.1 years). All patients had a clear history of trauma and presented with persistent pain and limited mobility. The left arm was involved in 4 cases and the right arm in 13 cases. The time from injury to surgery ranged from 1.5 to 24 months (mean 8.9 months). Postoperative follow-up ranged from 12 to 36 months (mean, 15.3 months), with no loss to follow-up in all patients. All surgeries included in this study were performed by two experienced pediatric orthopedic surgeons (with 15 and 12 years of clinical experience in pediatric upper limb trauma, respectively) at our institution. Both surgeons followed the same five-in-one surgical protocol throughout the study period.

## 3 Methods

### 3.1 Exposure and debridement of the humeroradial joint

**Figure 2:**
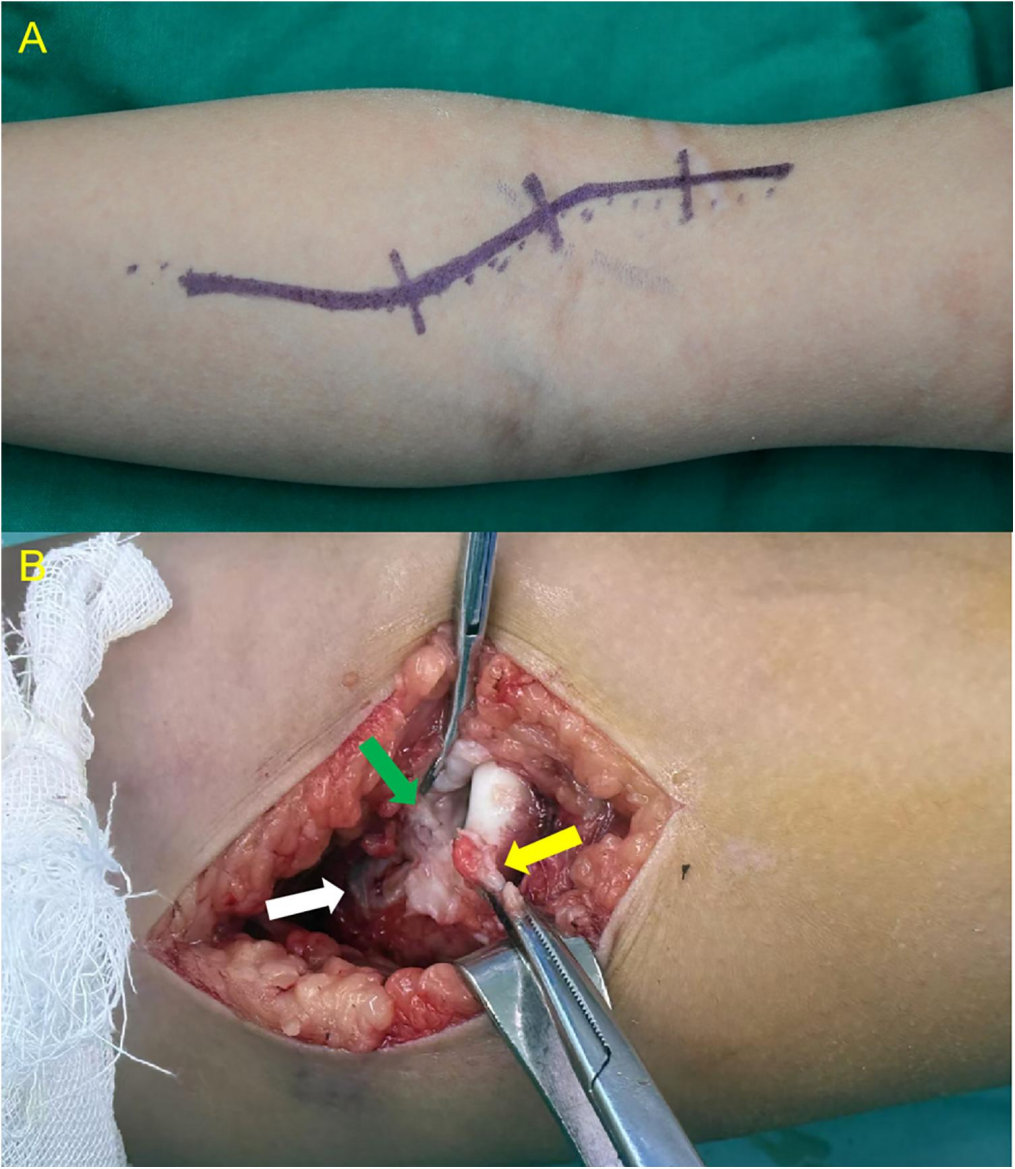
**(A)** Anterior surgical incision; **(B)** yellow arrow indicates the fibrous tissue covering the radial head. Green arrow points to the original anterior capsule of the humeroradial joint, which lies between the radial head and capitellum due to anterior dislocation. White arrow indicates the opened portion of the capsule.

The surgery was performed via the classic anterior *Henry* approach (Figure 2). A longitudinal incision was made on the anterolateral aspect of the elbow, starting 2 cm proximal to the elbow crease and extending distally along the lateral border of the biceps brachii, with a total length of approximately 8–10 cm. The skin, subcutaneous tissue, and deep fascia were incised sequentially. Care was taken to isolate and retract the superficial branch of the radial nerve, which often courses over the dislocated and prominent radial head. The prominent radial head was surrounded by fibrous connective tissue (not the joint capsule), which was incised to expose the radial head. Posterior to the radial head, a layer of dense fibrous tissue—representing the original anterior capsule of the humeroradial joint—was identified. This layer was opened longitudinally and extended proximally to expose the capitellum, and an attempt was made to pull this tissue out from behind the radial head. All obstructive scar tissue between the joint capsule and humeroradial joint was thoroughly debrided to eliminate mechanical barriers to radial head reduction.

### 3.2 Osteotomy and reduction

Reduction of the radial head was attempted but was often not achievable at this stage.A hinged external fixator was applied to the dorsal aspect of the ulna. A 1.5 cm longitudinal incision was made over the proximal ulna (approximately 1 cm distal to the radial tuberosity), and a transverse or short oblique osteotomy was performed. Lengthening and posterior angulation of the ulna were performed using the fixator's modules until complete reduction of the humeroradial joint was achieved. The hinge of the external fixator was locked after confirmation of humeroradial joint reduction. The stability of the humeroradial joint was assessed by performing elbow flexion-extension in forearm pronation, neutral, and supination positions; if instability was noted, the ulnar lengthening and angulation were readjusted immediately. The ulnar osteotomy site was percutaneously fixed with at least 2 K-wires (2 mm diameter) to enhance fixation stability and prevent fixator loosening.

### 3.3 Anterior joint capsule repair or augmentation

The surgical wound was thoroughly irrigated with normal saline. The incised anterior joint capsule was primarily sutured if possible; for patients with severe capsular contracture that could not be primarily closed, a biceps aponeurosis graft was harvested for capsular augmentation to restore the constraint function of the joint capsule. Annular ligament reconstruction was not performed in any patient.

### 3.4 Postoperative immobilization and rehabilitation

The wound was closed in layers, and a long-arm anterior plaster slab was applied to immobilize the elbow at 90° flexion and full forearm supination to maintain humeroradial joint reduction. Postoperative pin/K-wire infection may lead to fixation loosening and ultimately result in surgical failure, thus strict precautions should be taken to prevent postoperative infection. The plaster slab was removed at 6 weeks postoperatively, and elbow flexion-extension and forearm rotation exercises were initiated. The external fixator and K-wires were removed after radiographic confirmation of bridging callus at the osteotomy site.

## 4 Results

All surgeries were completed without intraoperative complications. No postoperative complications such as compartment syndrome or wound infection occurred. There were no iatrogenic nerve injuries. No pin/K-wire infection or loosening was found in any patient, and all ulnar osteotomy sites achieved bony union with no nonunion or delayed union.

**Figure 3:**
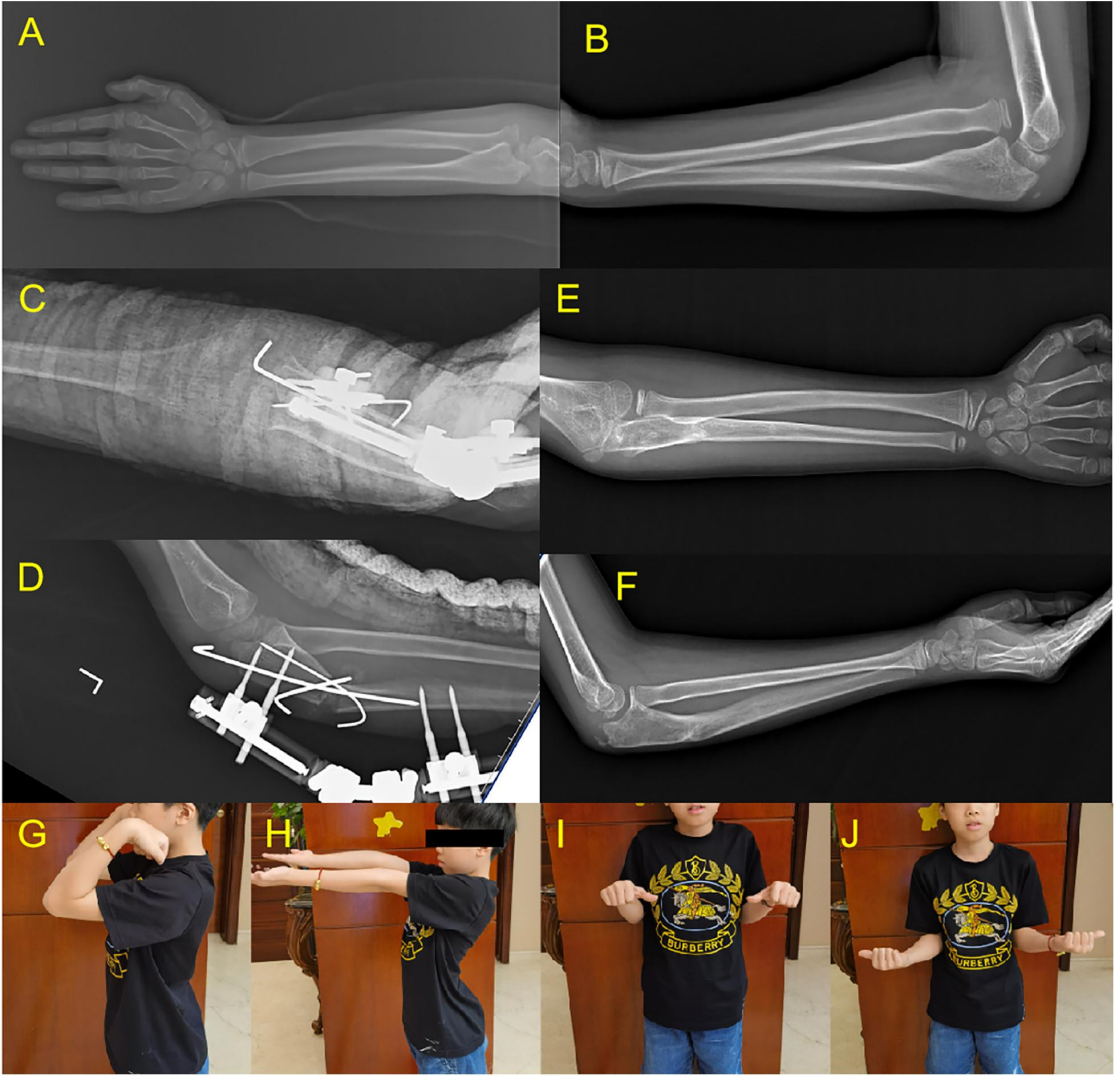
An 8-year-old male with left arm injury 12 months prior. **(A,B)** Preoperative X-rays showing anterior dislocation of the radial head. **(C,D)** Postoperative X-rays showing ulnar osteotomy with lengthening and angulation, fixed with external fixator, K-wires, and plaster slab. **(E,F)** X-rays at 1-year follow-up showing well-healed ulna and maintained humeroradial joint reduction. **(G–J)** Normal elbow flexion-extension, pronation, and supination at 1-year follow-up.

Two patients experienced redislocation of the radial head. In the remaining patients, follow-up X-rays at 6 months showed maintained reduction of the humeroradial joint, normal elbow flexion-extension, normal supination, mildly reduced pronation, no elbow valgus, and no pain (Figure 3).

**Figure 4:**
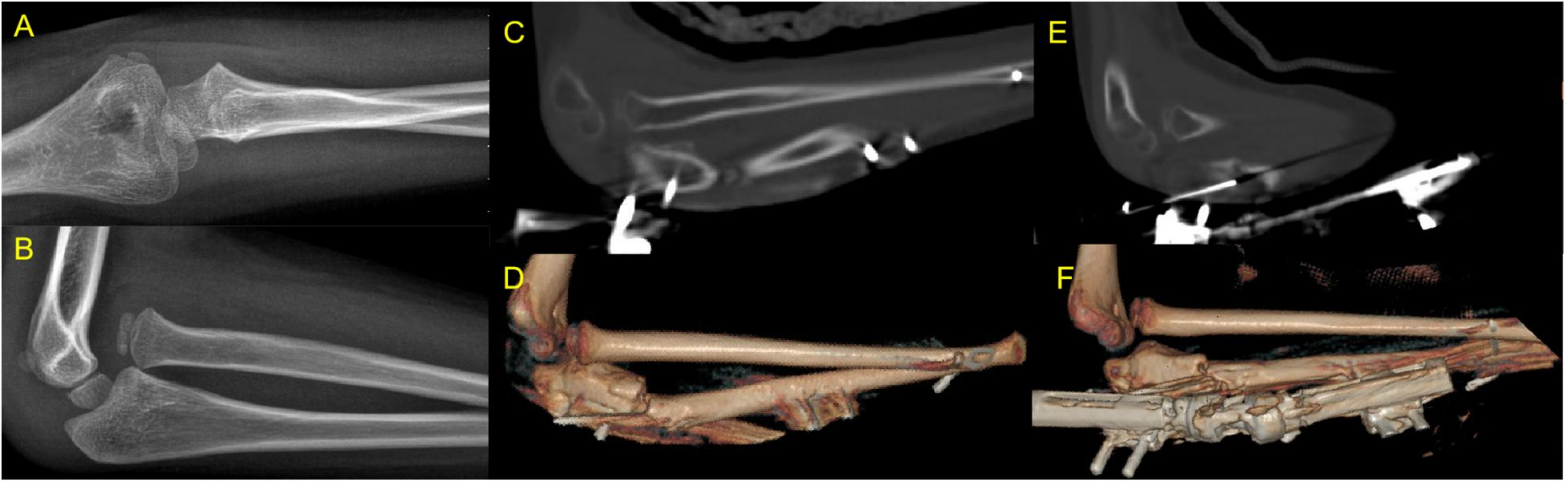
A surgical failure case with postoperative radial head redislocation. A 7-year-old male with elbow deformity 2 years after injury, showing radial head redislocation 1 month postoperatively. **(A,B)** Preoperative X-rays. **(C,D)** CT scan 2 days postoperatively showing reduced radial head. **(E,F)** CT scan 1 month postoperatively showing redislocation.

Anatomical reduction of the radial head was achieved in all patients intraoperatively. At the final follow-up, 15 patients (88.2%) maintained stable radial head reduction with no redislocation (Figure 3), while 2 patients (11.8%) experienced radial head redislocation at 1 month postoperatively (Figure 4). For these two redislocation cases, closed reduction and external fixator adjustment were attempted but failed to achieve stable humeroradial joint reduction, and the families declined further surgical intervention. The external fixator was removed after ulnar osteotomy healing, and no additional treatment was administered. At the 1-year follow-up, these two patients reported no elbow pain or discomfort, with preserved normal elbow range of motion consistent with preoperative status.

Four patients (23.5%) underwent biceps aponeurosis graft augmentation due to severe anterior joint capsule contracture that could not be primarily sutured; all four patients maintained stable radial head reduction at the final follow-up with no redislocation.

The elbow range of motion (ROM) was measured and compared between preoperative and 12 months postoperative (Table 1). Statistically significant improvements were observed in elbow flexion and extension function (flexion: 122.4 ± 12.5° vs. 126.5 ± 6.1°, *p* = 0.030; extension: -5.3 ± 7.2° vs. -2.9 ± 5.3°, *p* = 0.027). No statistically significant changes were found in forearm pronation and supination function (pronation: 84.7 ± 10.1° vs. 83.8 ± 6.5°, *p* > 0.05; supination: 85.6 ± 8.3° vs. 87.4 ± 5.0°, *p* > 0.05). All patients achieved functional recovery of the elbow with no significant limitation in daily activities at the final follow-up.

**Table 1:** Comparison of elbow range of motion before and 12 months after surgery (x ± s, °).

Elbow/forearm movement	Preoperative	Postoperative 12 months	*p*-value
Elbow flexion	122.4 ± 12.5°	126.5 ± 6.1°	0.030
Elbow extension	-5.3 ± 7.2°	-2.9 ± 5.3°	0.027
Forearm pronation	84.7 ± 10.1°	83.8 ± 6.5°	>0.05
Forearm supination	85.6 ± 8.3°	87.4 ± 5.0°	>0.05

## 5 Discussion

### 5.1 Pathological changes of chronic Monteggia fractures

The pathological changes in chronic Monteggia fractures include: (a) displacement of the radial head outside the joint capsule, with healed capsular tears forming a mechanical block to reduction and capsular contracture with intra-articular scar formation over time; (b) possible angulation deformity of the ulna; (c) relative overlengthening of the radius compared to the ulna; (d) deformation of the radial notch of the ulna, which may become shallow or disappear; and (e) degenerative changes in the articular surfaces of the radial head and capitellum, leading to loss of congruence. Except for ulnar angulation, the other four pathological changes worsen with longer dislocation duration, with the latter two being particularly challenging to address surgically. This explains why outcomes are less predictable in long-standing dislocations.

### 5.2 Analysis of surgical failure cases

The two cases of postoperative radial head redislocation were identified as surgical failures (Figure 4), and the root cause was confirmed as inadequate fixation of the ulnar osteotomy site: one patient received no K-wire supplementation and only external fixator fixation, and the other patient was fixed with only 1 K-wire plus the external fixator. Both patients developed hinged external fixator loosening during plaster immobilization, leading to loss of the corrected ulnar length and angulation, which ultimately caused radial head redislocation. No redislocation was observed in subsequent patients who received fixation with at least 2 K-wires plus the external fixator, confirming the importance of sufficient K-wire fixation for surgical success.

### 5.3 Key surgical decisions in the five-in-one approach

Anterior *Henry* approach for debridement and capsule repair: The anterior *Henry* approach was selected for Bado Type I chronic Monteggia fractures (anterior radial head dislocation) because it provides direct and sufficient exposure of the anterolateral elbow, facilitating thorough debridement of intra-articular scar tissue and the displaced anterior joint capsule—the main mechanical barriers to reduction. In addition, the anterior approach allows direct suture repair or augmentation of the anterior joint capsule after reduction, which functionally substitutes for the annular ligament to provide a circumferential constraint on the radial head and maintain humeroradial joint stability. Previous studies have reported the use of combined anterior-posterior approaches or posterior approaches for chronic Monteggia fractures, but these approaches are more invasive and not necessary for Bado Type I cases with anterior dislocation.

Hinged external fixator combined with at least 2 K-wires for fixation: Previous studies have described combined anterior-posterior approaches with plate fixation for ulnar osteotomy (17). Wang et al. proposed external fixator-assisted ulnar osteotomy followed by conversion to plate internal fixation for chronic Monteggia fractures (18), but the five-in-one approach abandons plate conversion for three reasons: The 1.5 cm mini-incision for ulnar osteotomy is minimally invasive and preserves the periosteal blood supply, promoting faster bony union; Plate conversion requires a larger surgical incision, increasing intraoperative trauma and medical costs; The combination of external fixator, K-wires, and postoperative plaster immobilization provides sufficient fixation stability, making plate internal fixation unnecessary. The two surgical failures in this study were due to inadequate K-wire fixation (0 or 1 K-wire), and subsequent cases with ≥2 K-wires had no redislocation, confirming that at least 2 K-wires are essential for augmenting fixator stability and should be included in the standardized surgical protocol.

Joint capsule repair/augmentation instead of annular ligament reconstruction: While some authors recommend annular ligament reconstruction (19), others consider it non-essential (20, 21). Annular ligament reconstruction is a traditional surgical procedure for Monteggia fractures, but it is not considered essential for pediatric Bado Type I chronic Monteggia fractures in this study. The repaired anterior joint capsule can functionally mimic the annular ligament to provide a stable circumferential constraint on the radial head, as confirmed by the stable reduction in all 4 patients who underwent biceps aponeurosis augmentation. Previous studies have also confirmed that annular ligament reconstruction is not a mandatory step for the treatment of pediatric chronic Monteggia fractures.

### 5.4 Five-in-one approach to other Bado types

This study exclusively included patients with Bado Type I chronic Monteggia fractures. For other Bado types, based on their distinct pathophysiological characteristics, the following possible surgical modifications may be considered: (a) Bado Type II (posterior radial head dislocation): a posterior approach is required for joint capsule debridement and repair (22), and the ulnar osteotomy should create an anterior angulation rather than posterior; (b) Bado Type III (lateral radial head dislocation): the ulnar osteotomy needs to be adjusted to correct medial angulation; (c) Bado Type IV (anterior radial head dislocation with both ulnar and radial shaft fractures): in addition to the five-in-one core steps, simultaneous fixation of the radial shaft fracture is required. We believe the core treatment principles for chronic Monteggia fractures remain constant: joint capsule debridement and repair, combined with external fixator and K-wire fixation of the ulnar osteotomy. It should be emphasized that the proposed modifications for Bado Types II, III, and IV are theoretical considerations, as no such cases were included in the present series. Future studies with larger sample sizes encompassing all Bado types are needed to further optimize individualized surgical protocols.

### 5.5 Strengths and limitations

This study introduces a novel standardized five-in-one surgical approach for the treatment of Bado Type I chronic Monteggia fractures, yielding promising initial results. The approach targets the reversible pathological changes associated with this condition and follows a clear and reproducible surgical protocol. Our findings demonstrate a high surgical success rate (88.2%) with no serious complications and satisfactory recovery of elbow function, thereby confirming the safety and effectiveness of this approach in clinical practice. Furthermore, by clearly identifying the root cause of surgical failure (inadequate K-wire fixation), we were able to optimize the surgical protocol accordingly, providing valuable clinical experience for its future application.

Despite these promising results, several limitations of this study must be acknowledged. First, the small sample size of only 17 patients limits the statistical power and makes it impossible to perform subgroup analyses (e.g., the effect of injury duration or age on surgical outcomes). Second, as a single-center retrospective design, the study is subject to selection bias, and no control group with traditional surgical methods was set up, making it impossible to conduct a comparative analysis of the efficacy of the five-in-one approach. Third, due to the exclusive inclusion of Bado Type I cases, the generalizability of the five-in-one approach to other Bado types of Monteggia fractures cannot be evaluated, and further studies with mixed Bado types are needed. Finally, the mean follow-up duration is only 15.3 months, and long-term follow-up (≥5 years) is needed to assess the long-term effects of the approach on pediatric elbow growth and development. Therefore, the findings should be interpreted with caution, and further multicenter prospective studies with larger cohorts and longer follow-up are warranted to validate the efficacy and safety of this approach.

In conclusion, successful treatment of chronic Monteggia fractures requires correction of the key pathological changes. The five-in-one surgery effectively addresses these issues. Except for two early failures due to inadequate fixation, all other cases achieved satisfactory outcomes, indicating that this approach is an effective surgical option.

## Data Availability

The original contributions presented in the study are included in the article/Supplementary Material, further inquiries can be directed to the corresponding author.
