# Supplementary material for: Five-in-one surgery: an integrated approach for chronic Monteggia fracture in children
Source: Front Surg. 2026 Mar 13;13:1797510. doi: 10.3389/fsurg.2026.1797510 (PMC13021401; doi:10.3389/fsurg.2026.1797510)
Supplement: Supplementary file 1 [file Datasheet1.pdf]

Supplementary Material

Supplementary Figures

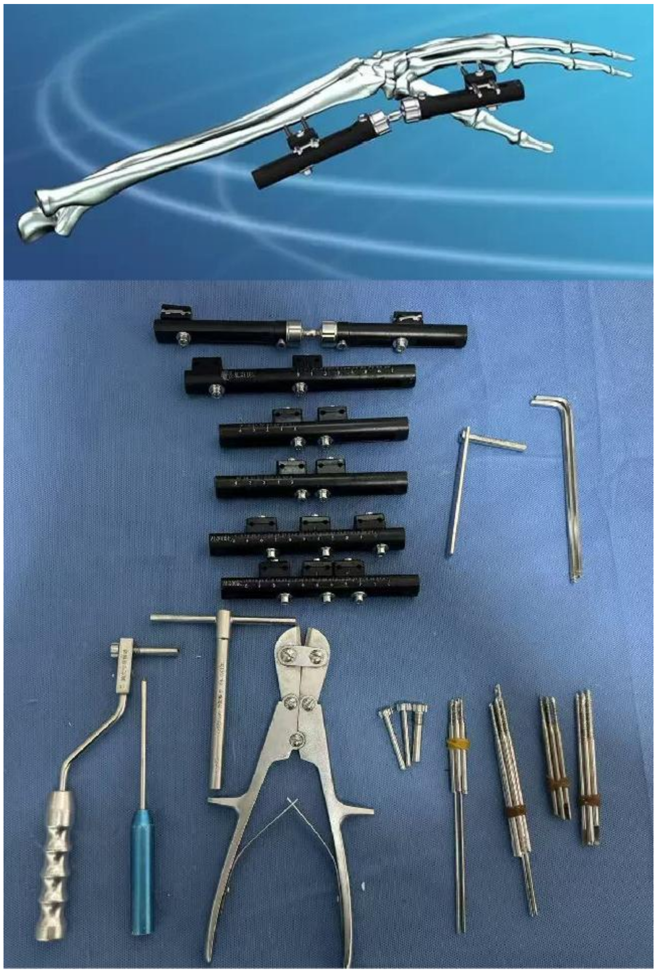

Supplementary Figure 1. The hinged external fixator used in this study.
